# Prevalence and associated factors of intestinal parasitic infections among patients attending Shahura Health Center, Northwest Ethiopia

**DOI:** 10.1186/s13104-019-4377-y

**Published:** 2019-06-11

**Authors:** Abiye Tigabu, Solomon Taye, Melak Aynalem, Kasaw Adane

**Affiliations:** 1 Department of Medical Microbiology, School of Biomedical and Laboratory Sciences, University of Gondar, P.O.BOX: 196, Gondar, Ethiopia; 2 Departement of Medical Laboratory Sciences, College of Medicine and Health Sciences, Wachamo University, Hossana, Ethiopia; 3 Department of Hematology and Immunohematology, School of Biomedical and Laboratory Sciences, University of Gondar, Gondar, Ethiopia; 4 Unit of Quality and Laboratory Management, School of Biomedical and Laboratory Sciences, University of Gondar, Gondar, Ethiopia

**Keywords:** Intestinal parasitic infections, Shahura Health Center, Intestinal helminths

## Abstract

**Objective:**

Parasitic infections are the commonest infections affecting 3.5 billion people leading 450 million illnesses. Parasites are major public health problems in developing countries. This study was aimed to assess the prevalence and associated factors of parasitic infections among patients. A cross sectional study was conducted on 364 patients, attending Shahura Health Center, Northwest Ethiopia. Stool specimens were collected and examined using formol-ether concentration technique. Socio-demographic data collected using questionnaire. Binary and multivariable logistic regression analyses were conducted to calculate the strength of association between variables.

**Result:**

The overall prevalence of intestinal parasitosis was 56.9%. The most prevalent parasite was *Entamoeba histolytica/dispar* 32.4% followed by *Hookworm* species 11.8% and *Giardia lamblia* 7.4% singly or mixed with other parasites. Furthermore, double and triple parasitic infections were observed in 3% and 1.4% patients respectively. Being male in gender (P = 0.049), age group interval between 1 and 20 years of old (P = 0.012), having stomach pain (P = 0.032) and having diarrhea (P = 0.007) were found to be significantly associated with parasitic infection. In conclusion, prevalence of parasitic infection in the area is high. Therefore, ensuring provision of clean potable water and minimizing the contamination of vegetables are recommended.

**Electronic supplementary material:**

The online version of this article (10.1186/s13104-019-4377-y) contains supplementary material, which is available to authorized users.

## Introduction

Intestinal parasitic infections are amongst the most common infections affecting approximately 3.5 billion people causing over 450 million ill health problems annually [1]. The ten global parasitic diseases are; *Amoebiasis*, *Ascariasis*, *Hookworm* infection and *Trichuriasis*. The three major soil-transmitted helminths of global health concern are; *Ascaris lumbricoides*, *Trichuris trichiura* and *Hookworm*. They cause over one billion infections and two billions are at risk of infection [2]. Furthermore, protozoan infections are another serious public health concerns and responsible for iron deficiency anemia, growth retardation, physical and mental health problems among children [3]. Infections with these parasites usually lead nutritional depletion, poor immunity in infants, mucosal loss and lymphatic leakage and local hemorrhage [4].

Parasitic infections affect the poorest and deprived communities of low and middle-income countries of the tropical and subtropical regions [5]. The main reasons for the high prevalence of parasite infections in tropical and subtropical countries were increasing population density, poor sanitation conditions, poor public health practices, inadequate toilet facilities, contaminated food and water, malnutrition, low host resistance and environmental changes [6].

Lack of safe water supplies and poor environmental sanitation situations are the main reasons for approximately 800 million expected cases of diarrheal incidences and the occurrence of 4.5 million mortalities in developing countries [7]. Behavioral, biological, environmental, socio-economic, health systems factors and local conditions such as quality of domestic and village infrastructure; household income, employment, occupational and social factors influence the risk of parasitic infections, disease transmission and associated morbidity and mortality [8]. Infection with pathogenic intestinal protozoa and helminths result in considerable morbidity, malnutrition and mortality worldwide, particularly among young children in developing countries and immune-compromised individuals [9–11].

In Ethiopia, the major health problems of Ethiopia are mainly preventable communicable parasitic diseases [12]. There are several reports which indicate the burden of parasitic diseases in Ethiopia. However, there are several localities including the current study area in which prevalence yet not determined. Therefore, this study was aimed to determine the prevalence of intestinal parasitic infections and associated factors among patients visiting Shahura Health Center, Northwest, Ethiopia.

## Main text

### Materials and methods

#### Study area

The study was conducted at Shahura town Health Center in Alefa Woreda, North Gondar Zone, Northwest, Ethiopia. The town is located 150 km Southwest of Gondar town at latitude of 12°361N and longitude of 37°281E with an elevation of 2133 m above sea level. In Shahura town (Alefa Woreda), the climate is warm and temperate. Alefa Woreda has 48 rural Kebeles with an estimated population of 240,000. In this district, majority of the fertile land is covered by cereals like teff, sorghum and maize. Permanent crops like coffee, hops and fruit trees are also grown. Most farmers both raise crops and livestock, while some only grow crops and very few only raise livestock. In addition there are also rivers crossing Shahura town and Alefa Woreda. Shahura is a small town with 58,441 dwellers. The town has one Health Center which provides different health services to the dwellers of Shahura town and surrounding rural Kebele populations.

#### Study design, population and sampling technique

A cross-sectional study design was used to assess the prevalence of intestinal parasitic infections and associated factors among patients visiting Shahura Health Center from June 2018 to September 2018. The study included patients who visited Shahura Health Center complaining gastrointestinal problems. Single population proportion formula was used to calculate the sample size. Study participants were consecutively enrolled until the targeted sample size three hundred sixty-four was achieved.

The aims of the study and benefits of participation were clearly explained to the participants prior to data collection. Participation was on voluntarily basis and they have told them it is their right to withdraw from the study any time during the course of data collection. A questionnaire based on known and possible factors was developed to explore the objectives of the study and pre-tested. Data’s on socio-demographic and associated factors were collected according to local culture and norm.

#### Stool specimen collection and examination

Fresh stool specimens were collected by experienced laboratory technologists who were selected and trained for the purpose of this study. Patients were instructed to collect the stool specimens into a leak-proof clean stool cup. All specimens were subjected to direct wet mount and formol-ether concentration technique [13]. A drop of normal saline or Lugol’s iodine and about 2 mg of stool were mixed on a microscopic glass slide, covered with cover slip and examined using microscope. After completion of direct stool examination, samples were emulsified in a 10% formalin solution and transported to University of Gondar, School of Biomedical and Laboratory Sciences Parasitology Laboratory to perform formol-ether concentration technique [13]. All standard procedures were strictly followed during stool sample examination to ensure the quality and sensitivity of the test result.

#### Data analysis

Data were checked for completeness and entered to EPI-Info version-7 and exported to SPSS version-20. The baseline characteristics of the study population were summarized using frequencies, mean and standard deviation. An internal comparison was made using binary logistic regression to determine the independent effect of the variables by calculating the strength of the association between IPIs and associated factors using odds ratio (OR) and 95% confidence interval (CI). Adjusted OR was computed using multivariable logistic regression to control the confounding variables. A *P* value less than 0.05 were considered statistically significant.

### Result

#### Socio-demographic characteristics

A total of 364 study participants were included in the present study. Of the total participants, 51.4% (187/364) were males while the remaining 48.6% (177/364) were females. Age of the study participants were ranged from 1 to 82 years with a mean age of 27.08 (SD + 12.851) years. Most of the study participants, 55.8% (203/364) were found in the age group of 18–30 years of old and nearly 60% (218/364) of the participants were rural residents. Among the study participants, 57.7% (210/364) had a family size fewer than five members and 36% (131/364) of the study participants were unable to read and write. Furthermore, majority of the study participants, 95.1% (346/364) were followers of Orthodox Christian religion and 79.1% (288/364) of the participants live with their family. Finally, 50.5% (184/364) of the participants were farmers in their profession (Additional file 1: Table S1).

#### Prevalence of intestinal parasitosis

**Fig. 1:**
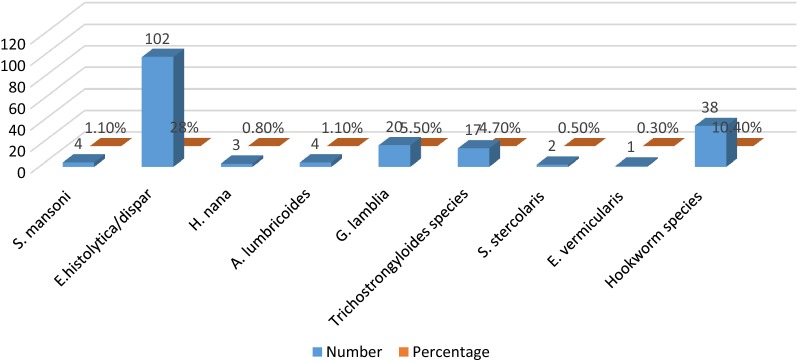
Prevalence of intestinal parasites by species among patients at Shahura Health Center, Northwest Ethiopia, 2018

Based on the microscopic examination of stool specimens, nine species of parasites were identified. Hence, the overall prevalence for at least one parasite is 56.9% (207/364). Of these detected parasites, *Entamoeba histolytica/dispar,* 32.4% (118/364) was the most common parasite followed by *Hookworm species* 11.8% (43/364), *G. lamblia* 7.4% (27/364), *Trichostrongyloides species* 4.7% (17/364), *A. lumbricoides* 2.2% (8/364) and *S. mansoni* 1.4% (5/364) singly or mixed with other parasites. Furthermore, double and triple parasitic infections were observed in 3% (11/364) and 1.4% (5/364) patients respectively. Additionally, one quadruple infection was detected (Table 1 and Fig. 1).

**Table 1:** Distribution of intestinal parasites species among patients (n = 364) at Shahura Health Center, Northwest Ethiopia, 2018

Type of intestinal parasite	Number of male +ve for IP (%)	Number of female +ve for IP (%)	Total number of +ve (%)
Single infection (n = 190)			
*E. histolytica/dispar*	61 (16.8)	41 (11.3)	102 (28)
*Hookworm species*	18 (4.9)	20 (5.5)	38 (10.4)
*G. lamblia*	9 (2.3)	11 (3)	20 (5.5)
*Trichostrongyloides species*	8 (2.2)	9 (2.3)	17 (4.7)
*A. lumbricoides*	3 (0.8)	1 (0.8)	4 (1.1)
*S. mansoni*	2 (0.5)	2 (0.5)	4 (1.1)
*H. nana*	3 (0.8)	0 (0.0)	3 (0.8)
*S. stercolaris*	1 (0.3)	1 (0.3)	2 (0.5)
Double infection (n = 11)			
*E. histolytica/dispar* and *G. lamblia*	3 (0.8)	2 (0.5)	5 (1.4)
*E. histolytica/dispar* and *A. lumbricoides*	1 (0.3)	2 (0.5)	3 (0.8)
*E. histolytica/dispar* and *H. nana*	1 (0.3)	1 (0.3)	2 (0.5)
*A. lumbricoides* and *Hookworm species*	1 (0.3)	0 (0.0)	1 (0.3)
Triple infection (n = 5)			
*E. histolytica/dispar, S. stercolaris* and *Hookworm species*	1 (0.3)	1 (0.3)	2 (0.5)
*E. histolytica/dispar, S. mansoni* and *Hookworm species*	2 (0.5)	0 (0.0)	2 (0.5)
*E. histolytica/dispar, G. lamblia* and *E. vermicularis*	1 (0.3)	0 (0.0)	1 (0.3)
Quadruple infection (n = 1)			
*E. histolytica/dispar, G. lamblia, S. mansoni* and *H. nana*	0 (0.0)	1 (0.3)	1 (0.3)
Total	115 (31.6)	92 (25.3)	207 (56.9)

The most prevalent intestinal parasites in double infection was *E. histolytica/dispar* and *G. lamblia* (5 patients; 1.4%), followed by *E. histolytica/dispar* and *A. lumbricoides* (3 patients; 0.8%); *E. histolytica/dispar* and *H. nana* (2 patients; 0.55%); and *A. lumbricoides* and *Hookworm species* (1 patients; 0.3%). Furthermore, 5 patients (1.4%) were infected by three species of parasites and one patient (0.3%) was parasitized by four species of parasites (*E. histolytica/dispar, G. lamblia, S. mansoni* and *H. nana*) (Table 1 and Additional file 2: Figure S1).

As it is depicted in Tables 1 and 2, 31.6% (115/364) of intestinal parasite infected participants were males. Highest intestinal parasitosis, 31.3% (114/364) was found among in the age groups 21–40 years of old followed by 1–20 years of old, 15.9% (58/364). The prevalence is higher in rural (131/364; 36%) than urban dwellers (76/364; 20.9%). Among the 207 infected patients, highest burden (112/364; 30.8%) of infection was found in patients who are illiterate and those who can read and write. Furthermore, intestinal parasitosis is high in patients those who have a family size between 1 and 5 (159/364; 43.7%).

**Table 2:** Factors associated with intestinal parasitosis among patients at Shahura Health Center, Northwest Ethiopia, 2018

Factors associated with intestinal parasitosis	Number (%)	Number of +ve for IP (%)	Number of −ve for IP (%)	Crude OR (95% CI)	P value	Adjusted OR (95% CI)	P value
Hand wash reason before meal							
Yes	313 (86)	181 (57.8)	132 (42.2)	0.758 [0.419–1.372]	0.361	–	–
No	51 (14)	26 (51)	15 (49)	1		–	–
Hand washing habit before meal							
Always	202 (55.5)	110 (54.5)	92 (45.5)	1.045 [0.273–4.007]	0.948	–	–
Mostly	23 (6.3)	12 (52.2)	11 (47.8)	1.146 [0.244–5.391]		–	–
Often	6 (1.6)	1 (16.7)	5 (83.3)	6.250 [0.504–77.494]		–	–
Sometimes	124 (34.1)	79 (63.7)	45 (36.3)	0.712 [0.182–2.788]		–	–
Rarely	9 (2.5)	5 (55.6)	4 (44.4)	1		–	–
Source of drinking water							
Pipe	173 (47.5)	94 (54.3)	79 (45.7)	1.513 [0.908–2.520]	0.112	–	–
River	56 (15.4)	32 (57.1)	24 (42.9)	1.350 [0.690–2.642]		–	–
Pond	37 (10.2)	18 (48.6)	19 (51.4)	1.900 [0.883–4.086]		–	–
Unprotected spring	98 (26.9)	63 (64.3)	35 (35.7)	1		–	–
Storage of water in wide-open pot							
Yes	108 (29.7)	60 (55.6)	48 (44.4)	1.079 [0.686–1.698]	0.743	–	–
No	256 (70.3)	147 (57.4)	109 (42.6)	1		–	–
Contact with water bodies							
Yes	73 (20.1)	42 (57.5)	31 (42.5)	0.967 [0.575–1.624]	0.898	–	–
No	291 (79.9)	165 (56.7)	126 (43.3)	1		–	–
Habit of eating raw vegetable							
Yes	97 (26.6)	60 (61.9)	37 (38.1)	0.755 [0.470–1.215]	0.248	–	–
No	267 (73.4)	147 (55.1)	120 (44.9)	1		–	–
Habit of eating raw meat							
Yes	98 (26.9)	58 (59.2)	40 (40.8)	0.878 [0.549–1.405]	0.588	–	–
No	266 (73.1)	149 (56)	117 (44)	1		–	–
Have latrine							
Yes	316 (86.8)	182 (57.6)	134 (42.4)	0.800 [0.435–1.471]	0.473	–	–
No	48 (13.2)	25 (52.1)	23 (47.9)	1		–	–
Type of latrine							
Family	299 (82.1)	174 (58.2)	125 (41.8)	0.678 [0.336–1.368]	0.279		
Public	30 (8.2)	16 (53.3)	14 (46.7)	0.826 [0.311–2.195]		–	–
Field	35 (9.7)	17 (48.6)	18 (51.4)	1		–	–
Latrine usage habit							
Always	223 (61.3)	124 (55.6)	99 (44.4)	0.748 [0.353–1.588]	0.450	–	–
Sometimes	110 (30.2)	68 (61.8)	42 (38.2)	0.579 [0.260–1.292]		–	–
Rarely	31 (8.5)	15 (48.4)	16 (51.6)	1		–	–
Hand washing habit after toilet visit							
Always	171 (47)	90 (52.6)	81 (47.4)	1.050 [0.523–2.109]	0.891	–	–
Mostly	25 (6.9)	14 (56)	11 (44)	0.917 [0.334–2.517]		–	–
Often	19 (5.2)	14 (73.7)	5 (26.3)	0.417 [0.126–1.383]		–	–
Sometimes	110 (30.2)	68 (61.8)	42 (38.2)	0.721 [0.345–1.507]		–	–
Rarely	39 (10.7)	21 (53.8)	18 (46.2)	1		–	–
Presence of domestic animal							
Yes	234 (64.3)	139 (59.4)	95 (40.6)	1.334 [0.866–2.055]	0.191	–	–
No	130 (35.7)	68 (52.3)	62 (47.7)	1		–	–
Presence of cat or dog							
Yes	216 (59.3)	134 (62)	82 (38)	0.596 [0.390–0.910]	0.016	–	–
No	148 (40.7)	73 (49.3)	75 (50.7)	1		–	–
Presence of stomach pain							
Yes	337 (92.6)	196 (58.2)	141 (41.8)	0.455 [0.223–0.989]	0.048	0.401 [0.174–0.925]	0.032
No	27 (7.4)	11 (40.7)	16 (59.3)	1		1	
Presence of diarrhea							
Yes	170 (46.7)	89 (52.4)	81 (47.6)	0.413 [0.931–0.844]	0.041	0.474 [0.191–0.948]	0.007
No	194 (53.3)	118 (60.8)	76 (39.2)	1		1	
Presence of bloody diarrhea							
Yes	42 (11.5)	27 (64.3)	15 (35.7)	0.704 [0.361–1.374]	0.304	–	–
No	322 (88.5)	180 (55.9)	142 (44.1)	1		–	–
Habit of trimming nail							
Yes	289 (79.4)	169 (58.5)	120 (41.5)	0.729 [0.438–1.214]	0.225	–	–
No	75 (20.6)	38 (50.7)	37 (49.3)	1		–	–
Presence of dirt on the nail							
Yes	131 (36)	81 (61.8)	50 (38.2)	0.727 [0.470–1.125]	0.152	–	–
No	233 (64)	126 (54.1)	107 (45.9)	1		–	–
Have protective shoes							
Yes	341 (93.7)	194 (56.9)	147 (43.1)	0.985 [0.420–2.309]	0.972	–	–
No	23 (6.3)	13 (56.5)	10 (43.5)	1		–	–
Habit of wearing shoes							
Always	230 (63.2)	122 (53)	108 (47)	1.012 [0.355–2.882]	0.983	–	–
Sometimes	119 (32.7)	77 (64.7)	42 (35.3)	0.623 [0.211–1.839]		–	–
Rarely	15 (4.1)	8 (53.3)	7 (46.7)	1		–	–
Personal hygiene							
Good	262 (72)	143 (54.6)	119 (45.4)	1.402 [0.877–2.241]	0.159	–	–
Poor	102 (28)	64 (62.7)	38 (37.3)	1		–	–

Regarding the study variables, 86% (313/364) of them know why they wash their hands before meal and 55.5% (202/364) of the participants are always wash their hands before eating; 70.3% (256/364) do not put water in wide-open pot or plastic container, and 73.4% (267/364) and 73.1% (266/364) of the participants were do not have habit of eating raw vegetables and meat, respectively. In addition, 47.5% (173/364) of the study participants were obtain drinking water from pipe. Study participants those had latrine are 86.8% (316/364) and only 61.3% (223/364) of them are always use latrine. Among the study participants, 79.4% (289/364) have habit of trimming their finger nail regularly. Furthermore, 93.7% (341/364) of them have shoes and only 63.2% (230/364) of them are always wear their shoes. In this study, 92.6% (337/364) of the participants had stomach pain and 46.7% (170/364) of the patients were diarrheic. Finally, 64.3% (234/364) of study participants have at least one domestic animal and 59.3% (216/364) have at least one pet (Table 2).

The distribution of parasites among study variables were shown in Table 2. Hence, 16.5% (60/364) of the participants those have habit of eating raw vegetables and raw meat 15.9% (58/364) were infected by parasites. Of the participants, 50% (182/364) positive cases have latrine at their home. Furthermore, 53.8% (196/364) of the positive patient cases had stomach pain and 24.5% (89/364) of parasite infected patients were diarrheic.

#### Factors associated with intestinal parasites

In this study several factors were considered to assess factors that contribute to intestinal parasite infection. Binary and multivariable logistic analyses were calculated. Multivariable logistic analysis was conducted after adjusting variables which were statistically significant in binary logistic analysis and from the factors associated with intestinal parasites; being male in gender (P = 0.049; AOR = 0.645; CI 0.416–0.998), age group interval between 1 and 20 years of old (P = 0.012; AOR = 0.645; CI 0.458–0.908), having stomach pain (P = 0.032; AOR = 0.401; CI 0.174–0.925) and having diarrhea (P = 0.007; AOR = 0.474; CI 0.191–0.948) were found to be significantly associated with parasitic infections. However, majority of the study variables were non-significantly associated with intestinal parasitic infections with a P-value of > 0.05 (Table 2).

### Discussion

Intestinal parasitic infections are distributed virtually throughout the world and results in an impact on human health and development [1]. Developing countries are more affected by parasites than bacterial infections [4]. In this study, the overall prevalence of intestinal parasitosis was 56.9%. The finding of this study was higher than prevalence of parasites at Workmeda Health Center, Ethiopia (27.7%), Uganda (32.8%), Nigeria (41.2%), India (49.38%) and United Arab Emirates (7.74%) [14–19]. On the other hand, this study had showed a lower prevalence of parasitic infection than the reports in Southwest Ethiopia (83%), Tseda Health Center, Northwest Ethiopia (62.2%) and India (92.32%) [9, 20–22]. These variations might be due to the difference in the characteristics of the study population, geographical distribution and diagnostic techniques.

In this study, among the 207 infected patients, 115/364 (31.6%) were males which indicated that higher proportion of intestinal parasitic infections among male than female patients, 92/364 (25.3%). The higher prevalence in males might be due to everyday participation of males in outdoor activities is than females which make them more vulnerable to parasitic infections. Intestinal parasitic infections were higher in rural 36% (131/364) than urban patients 20.1% (76/364). This might be due to poor personal hygiene, over-crowding and the frequent contamination of water bodies and animals in rural than urban community.

The prevalence of *E. histolytica/dispar* in this study is higher when compared to other detected intestinal parasites. The possible reason might be due to contamination of potable water, poor handling of food stuff, contamination of food, and eating food without washing hands. The prevalence of intestinal parasites in direct wet mount and formol-ether sedimentation technique was quite different. This variation of prevalence of intestinal parasites in direct wet mount and formol-ether sedimentation technique might be due to the difference in the characteristics of intestinal parasites; more trophozoite stages of protozoan parasite detected in direct wet mount technique but not in formol-ether sedimentation technique. It might be due to the highly fragile nature of the trophozoite and the effect of preservatives in formol-ether sedimentation technique.

Several studies showed that socio-demographic characteristics and associated factors contribute a lot to contract intestinal parasitic infections [13, 23, 24]. The result of this study showed that being male in gender was significantly associated with intestinal parasitic infections. The possible reason might be involvement of males in farming activities than females which exposes them to more animal contact, water body contact and soil contamination. Age category of study subjects belonging to 1–20 years of old was a significant factor for intestinal parasitic infections which showed that being young in age, the more likely to develop intestinal parasitic infections. The possible reasons might be due to frequent contact among each other, playing with soil and water bodies, over-crowding in classroom or day-care centers, poor personal hygiene, immuno-compromised condition and habit of sharing of materials which facilitates the spread of the parasites.

Stomach pain in this study was a statistically significant factor to intestinal parasitic infections. Our study showed that study participants having stomach pain were more likely to be positive for parasites than those who have no stomach pain. The possible reason might be due to the abdominal cramps, bloating, nausea and watery diarrhea during parasitic infections. Most intestinal parasitic infections were associated with diarrhea. In this study having diarrhea also was significantly associated with parasitic infections. The possible reason might be due to diarrhea causing nature of intestinal parasites.

### Conclusion

The prevalence of intestinal parasitic infection in our study was 56.9%. Being male in gender, age less than 20 years, having stomach pain and having diarrhea were associated factors for intestinal parasite infection. Ensuring provision of clean potable water and minimizing the contamination of vegetables are recommended. Furthermore, health education on wearing protective shoe and deworming of carriers should also be done. Finally, large community-based studies assessing all associated risk factors and continuous surveillance should be conducted to halt the spread of intestinal parasite infections in Shahura town and Alefa Woreda.

## Limitations of the study

Due to resource constraints, we did not perform molecular techniques like PCR to identify and discriminate the true pathogenic *E. histolytica from E. dispar*. Thus, over diagnosis of Amoebiasis might occur. And also we did not perform other sensitive methods specific for some intestinal parasites such as Kato-Katz method, Trichome and modified Ziehl–Neelsen staining methods.

## Additional files

**Additional file 1: Table S1.** Socio-demographic characteristics of study participants among patients at Shahura Health Center, Northwest Ethiopia, 2018.

**Additional file 2: Figure S1.** The overall burden of intestinal parasites in terms of their frequency per individual among patients at Shahura Health Center, Northwest Ethiopia, 2018.

## Data Availability

All data generated or analyzed during this study are included in this article. Data that support the findings of this study are also available from the corresponding author upon reasonable request.

## Abbreviations

AIDS: Acquired Immune Deficiency Syndrome
CI: confidence interval
GIT: gastrointestinal tract
GOs: Governmental Organizations
HIV: human immunodeficiency virus
IPs: intestinal parasites
IPIs: intestinal parasitic infections
NGOs: Non-Governmental Organizations
OR: odds ratio
SPSS: Statistical Package of Social Science
WHO: World Health Organization
