# Supplementary material for: Prevalence and associated factors of intestinal parasitic infections among patients attending Shahura Health Center, Northwest Ethiopia
Source: BMC Res Notes. 2019 Jun 11;12:333. doi: 10.1186/s13104-019-4377-y (PMC6560869; doi:10.1186/s13104-019-4377-y)
Supplement: Supplementary file 1 — Additional file 1: Table S1. Socio-demographic characteristics of study participants among patients at Shahura Health Center, Northwest Ethiopia, 2018. [file 13104_2019_4377_MOESM1_ESM.docx]

**Table S1:** Socio-demographic characteristics of study participants among patients at Shahura health center, Northwest Ethiopia, 2018.

| Characteristics | | Number (%) | Number of positive for IP (%) | Number of negative for IP (%) | Crude OR (95% CI) | P value | Adjusted OR (95% CI) | P value |
| --- | --- | --- | --- | --- | --- | --- | --- | --- |
| Gender | Male | 187 (51.4) | 115 (61.5) | 72 (38.5) | 0.648 [0.427-0.983] | 0.041* | 0.645 [0.416-0.998] | **0.049*** |
|  | Female | 177 (48.6) | 92 (52) | 85 (48) | 1 |  | 1 |  |
| Age group interval | 1-20 years | 123 (33.8) | 58 (47.2) | 65 (52.8) | 0.664 [0.474-0.954] | 0.003* | 0.645 [0.458-0.908] | **0.012*** |
|  | 21-40 years | 191 (52.5) | 114 (59.7) | 77 (40.3) | 0.872 [0.433-0.752] | 0.679 | 0.797 [0.433-0.975] |  |
|  | > 40 years | 50 (13.7) | 35 (70) | 15 (30) | 1 |  | 1 |  |
| Residence | Rural | 218 (59.9) | 131 (60.1) | 87 (39.9) | 0.721 [0.472-1.101] | 0.130 | - | - |
|  | Urban | 146 (40.1) | 76 (52.1) | 70 (47.9) | 1 |  | - | - |
| Living situation | With family | 288 (79.1) | 174 (60.4) | 114 (39.6) | 0.568 [0.202-45.400] | 0.910 | - | - |
|  | Rent | 75 (20.6) | 32 (42.7) | 43 (57.3) | 0.761 [0.369-99.324] |  | - | - |
|  | Other | 1 (0.3) | 1 (100) | 0 (0) | 1 |  | - | - |
| Family size category | 1-5 | 274 (75.3) | 159 (58) | 115 (42) | 0.936 [0.854-1.026] | 0.159 | - | - |
|  | >5 | 90 (24.7) | 48 (53.3) | 42 (46.7) | 1 |  | - | - |
| Religion | Orthodox | 346 (95.1) | 196 (56.6) | 150 (43.4) | 0.703 [0.121-9.833] | 0.683 | - | - |
|  | Muslim | 16 (4.3) | 9 (56.3) | 7 (43.7) | 0.734 [0.018-78.205] |  | - | - |
|  | Protestant | 1 (0.3) | 1 (100) | (0) | 0.980 [0.873.12.042] |  | - | - |
|  | Other | 1 (0.3) | 1(100) | (0) | 1 |  | - | - |
| Educational status | Illiterate | 131 (36) | 74 (56.5) | 57 (43.5) | 1.455 [0.746-2.837] | 0.271 | - | - |
|  | Read and write | 60 (16.5) | 38 (63) | 22 (37) | 1.094[0.503-3.376] |  | - | - |
|  | Primary school | 50 (13.7) | 28 (56) | 22 (44) | 1.484 [0.668-3.299] |  | - | - |
|  | Secondary school | 71 (19.5) | 33 (46.5) | 38 (53.5) | 2.175 [1.040-4.547] |  | - | - |
|  | Above grade 12 | 52 (14.3) | 34 (65.4) | 18 (34.6) | 1 |  | - | - |
| Occupation | Government employee | 55 (15.1) | 32 (58.2) | 23 (41.8) | 0.958 [0.195-4.699] | 0.958 | - | - |
|  | Non-government employee | 5 (1.4) | 3 (60) | 2 (40) | 0.889 [0.086-9.162] |  | - | - |
|  | Farmer | 184 (50.5) | 114 (62) | 70 (38) | 0.819 [0.178-3.767] |  | - | - |
|  | Merchant | 29 (8) | 14 (48.3) | 15 (51.7) | 1.429 [0.270-7.549] |  | - | - |
|  | Student | 80 (22) | 36 (45) | 44 (55) | 1.630 [0.342-7.758] |  | - | - |
|  | Unemployed | 4 (1) | 4 (100) | 0 (0) | 0.798 [0.651-8.763] |  | - | - |
|  | Other | 7 (2) | 4 (57.1) | 3 (42.9) | 1 |  | - | - |
