# Supplementary material for: Prevalence and associated factors of intestinal parasitic infections among patients attending Shahura Health Center, Northwest Ethiopia
Source: BMC Res Notes. 2019 Jun 11;12:333. doi: 10.1186/s13104-019-4377-y (PMC6560869; doi:10.1186/s13104-019-4377-y)

Supplementary files

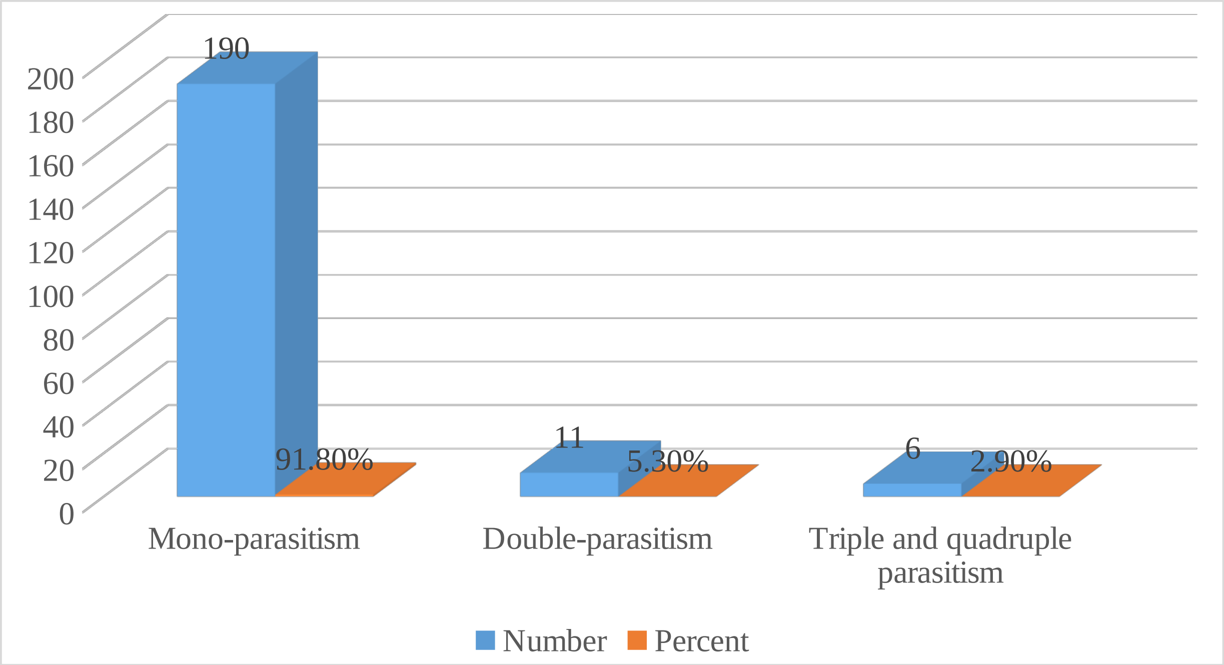

Supplement: Supplementary file 2 [file 13104_2019_4377_MOESM2_ESM.docx]
